# *In Vivo* Response of Acellular Porcine Pericardial for Tissue Engineered Transcatheter Aortic Valves

**DOI:** 10.1038/s41598-018-37550-2

**Published:** 2019-01-31

**Authors:** Reza Khorramirouz, Jason L. Go, Christopher Noble, David Morse, Amir Lerman, Melissa D. Young

**Affiliations:** Department of Cardiovascular Medicine, Mayo Clinic, Rochester, MN USA

## Abstract

Current heart valve prostheses have limitations that include durability, inability to grow in pediatric patients, and lifelong anticoagulation. Transcatheter aortic valve replacements are minimally invasive procedures, and therefore have emerged as an alternative to traditional valve prostheses. In this experiment, the regenerative capacity of potential tissue engineered transcatheter valve scaffolds (1) acellular porcine pericardium and (2) mesenchymal stem cell-seeded acellular porcine pericardium were compared to native porcine aortic valve cusps in a rat subcutaneous model for up to 8 weeks. Immunohistochemistry, extracellular matrix evaluation, and tissue biomechanics were evaluated on the explanted tissue. Acellular valve scaffolds expressed CD163, CD31, alpha smooth muscle actin, and vimentin at each time point indicating host cell recellularization; however, MSC-seeded tissue showed greater recellularization. Inflammatory cells were observed with CD3 biomarker in native porcine pericardial tissue throughout the study. No inflammation was observed in either acellular or MSC-seeded scaffolds. There was no mechanical advantage observed in MSC-seeded tissue; however after the first week post-explant, there was a decrease in mechanical properties in all groups (p < 0.05). MSC-seeded and acellular porcine pericardium expressed decreased inflammatory response and better host-cell recellularization compared to the native porcine aortic valve cusps.

## Introduction

Valvular heart disease (VHD) is a prevalent and life altering condition that currently has limited treatment options. In industrialized countries, the prevalence of valvular heart disease is estimated at 2.5%^1^. Treatment options require heart valve replacement, which are commonly limited to mechanical or bioprosthetic valves and although autologous valvular implants are most desirable for host cell integration and biocompatibility, they are neither readily available nor cost effective^2–4^. For younger patients the Ross procedure, where the patient’s pulmonary valve is grafted in the aortic position and a replacement valve is placed in the now vacant pulmonary position, has shown good long term outcomes^5,6^. Also the Ozaki technique where the aortic valve is reconstructed from glutaraldehyde treated autologous pericardium, or decellularized allografts, are both further feasible alternatives^7–9^. Finally the David operation, where aortic cusps are sutured onto an artificial rigid root to spare the valves when aortic root repair is necessary is an alternative in this circumstance with strong outcomes^10^. However in general, transcatheter aortic valve replacements (TAVR) are replacing open heart procedures because of decreased mortality (barring cases of severe paravalvular leakage), blood loss, and hospital stays^2–4^.

Research on valvular heart repair has focused on tissue-engineered heart valves (TEHV) because of its similarities to native heart valves^11^. Current options for TEHV include synthetic and xenograft tissue; however, these prosthetics have been associated with increased inflammation, infection, thrombogenecity, and calcification risk^12^. Alternatively, decellularized human tissue for TEHV has shown good results but sourcing tissue is problematic^13–15^. Decellularization of xenograft tissue is a promising approach because it elicits less immune response with increased recellularization capacity serving as a biologic scaffold for cell attachment, migration, and proliferation^16–18^. Host cell invasion and subsequent remodeling allows the implanted valve to grow and adapt with the patient. Clinical applications of TEHV require extensive testing to ensure long term compatibility, recellularization, remodeling, and mechanical performance. In preliminary studies, our team has engineered a TEHV utilizing acellular pericardial tissue that showed superior mechanical behavior compared to native valve cusps^19^. The TAVR was created by suturing decellularized and sterilized porcine pericardial tissue onto a self-expanding nitinol stent for transcatheter delivery. In previous studies, we have shown that our decellularization and sterilization process produced a stronger and more resilient tissue^20^. This processed tissue was able to recellularize with host cells after 5-months implantation in an ovine model^18^.

Introduction of *in vitro* seeding using mesenchymal stem cells (MSCs) has been investigated for its potential to aid in recellularization and remodeling. MSCs are considered to be immunoprivileged because they express very low levels of MHC class I and II^21^. The current study evaluated the *in vivo* behavior of acellular porcine pericardium, MSC-seeded porcine pericardium, and native porcine aortic valve cusps in an *in vivo* model. Subcutaneous implantation in a rat model was utilized to evaluate immune reaction, recellularization, and biomechanical properties. We hypothesized that acellular and MSC-seeded porcine pericardium has better regenerative capacity with minimal inflammation compared to native porcine aortic valve cusps.

## Materials and Methods

### Procurement, decellularization, and sterilization of porcine pericardium and aortic valve cusps

Porcine pericardium and aortic valves were obtained from a local abattoir (Hormel Food Corporation, USA). The tissue was cleaned and washed with phosphate buffered saline solution (PBS). The pericardium and aortic valve was decellularized with 0.5% sodium dodecyl sulfate (SDS) (Invitrogen Cat No. 15525017) in diH_2_O with DNase Grade II (Roche 10104159001) for 48 hours in 4 °C while agitating. The tissue was rinsed for 48 hours with 2% DNase, Tris buffer, and MgCl_2_. The tissue was washed in PBS & 1% antibiotic/antimycotic for 14–21 days, changing the solution once daily, using agitator in 4 °C^18^. Supercritical carbon dioxide (NovaSterilis, Inc., USA) was used for sterilization as described in our previous work^18,20,22^.

### *In vitro* recellularization with mesenchymal stem cell (MSC)

Green fluorescent protein (GFP)-tagged rat bone marrow–derived MSCs (Cyagen, USA) at passage number 3 was seeded on the acellular porcine pericardium. The cell seeding density was 40,000 cells/sq. cm. The tissue sample area was 4 sq. cm. and thus, 200,000 cells in 50 µl cell culture media were seeded on each tissue sample. After cell seeding, the tissue samples were placed in a cell incubator (at 37 °C and 5% CO_2_) for half hour for cell attachment to the tissues. Then, 2-mL cell culture media was added to each cell-attached tissue sample, which was then cultured for 24-hours before its implantation in a rat. Cell viability was assessed using a LIVE/DEAD® Assay (Thermofisher Scientific, Waltham, Massachusetts, USA) per manufacturer guidelines.

### Surgical procedure

 Nine Sprague Dawley rats were obtained and kept in a pathogen free environment. Decellularized and sterilized porcine pericardium, with and without seeded MSCs, were implanted subcutaneously in a rat dorsum and explanted after 1, 4, and 8 weeks. The animals were divided into 3 groups, each rat had 2 implants, and the tissue engineered implants were large, which enabled us to obtain 6 specimens per time point (or 18 specimens per group, for a total of 54 specimens). This sample size (n = 18 per group) was sufficient for the statistical analysis performed, and provided power >0.80. The groups were as follows: Group 1: Native porcine aortic valve cusps (n = 6), Group 2: Acellular porcine pericardium (n = 6), Group 3: GFP-positive mesenchymal stem cells (MSCs) seeded on acellular porcine pericardium (n = 6). Tissue from all 3 groups were sutured and fixed onto a biocompatible polycaprolactone (PCL, Mn 45,000, and Sigma-Aldrich) frame using polypropylene 5–0. This methodology was previously used to successfully evaluate tissue engineered scaffolds^23^. The rats were induced by inhalant isoflurane 4%, maintained at 2%, and then subcutaneous buprenorphine 0.6 mg/kg. The animals were positioned in a prone position and a 2 cm dorsal midline incision was made over the thoracolumbar area. The skin was released by adjacent fascia, and the frame was placed beneath the skin. The distance between the implant pockets was approximately 5 cm. Skin closure was achieved using Vicryl 4–0 and animals received tetracycline ointment. At explant, samples were biopsied after 1, 4 and 8 weeks post-operation.

### Histology and immunohistochemistry

Explanted tissue from each group was removed, paraffin-embedded, and sectioned. Different antibodies were used for immunohistochemical (IHC) staining to determine angiogenesis, host cell recellularization, and immune reaction. Biomarkers CD31/CD34 were applied for angiogenesis, α-smooth muscle actin (α-SMA)/vimentin determined the presence of interstitial-like cell phenotypes, and CD3/CD163 were used to evaluate immune reaction. Tissue samples were labeled with the Dako Envision System-HRP, blocked with the Dako peroxidase solution (Aligent, Santa Clara, CA), incubated in primary antibody overnight at 4 °C, and stained with a species-matched secondary antibody (biotinylated rabbit anti-rabbit Ig F [ab’]2 fragments; Dako). The bound antibody was visualized using Sigma Fast 3,3-diaminobenzidine (DAB) and sections were counterstained, dehydrated, and mounted. All the images were captured using light and polarized microscopy, and the area of antibody signal was quantified using Image J 1.48n software (National Institutes of Health, Bethesda, MD)^24^. Analysis was performed using the Threshold analysis, by using the option Measure to calculate the area fraction on the examined sections. The percentage of IHC staining was calculated by dividing the IHC stained area over the total area. The GFP-positive pericardium was visualized under fluorescent microscope after explantation to observe cellularization in the acellular pericardium. In addition, samples underwent Hematoxylin-Eosin (H&E), 4,6-Diamidino-2-Phenylindole Dihydrochloride (DAPI), Picrosirius red, and Masson Trichrome stains to observe DNA, cellularity, fibrosis, and collagen deposition; respectively.

### Transmission electron microscopy (TEM)

Scaffolds were stored in 4 °C for 24-hours, and then rinsed in PBS. After storage, the tissue was fixed in 1% osmium tetroxide (OsO4), dehydrated through a graded series of ethanol, and embedded in Spur resin. Sections were cut with an ultra-microtome (100 nm or 0.1μm), stained in 3% (w/v) uranyl acetate in 70% (v/v) EtOH for 20 min and in Reynold’s lead citrate for 20 min. Imaging was captured using the JEOL “JEM-1400 Plus” transmission electron microscope.

### Biomechanical properties of scaffolds

The uniaxial tensile testing was conducted for each group (n = 6 specimens per group, n = 18 for all groups) after 1, 4 and 8 weeks (Instron, USA). Collagen fibers were aligned circumferentially and each group was cut into 4 equal pieces with dimensions of 6 mm × 10 mm. The thickness was measured using a thickness gauge (Mitutoyo, Japan). Samples were loaded at the extension rate 0.1667 mm/s per ASTM standards (ASTM F2150-13) and stretched until failure^25^. Tensile stress was calculated by dividing the measured force by the initial cross sectional area and strain by dividing the measured displacement by the initial sample length.

### Statistical analysis

Data distribution was analyzed utilizing MATLAB statistics toolbox using a one way ANOVA followed by a multi-comparison test. Data is presented as mean ± SD with statistical significance defined as *P* value < 0.05.

### Animal studies

The authors confirm that all methods were carried out in accordance with relevant guidelines and regulations, and standard of care was approved by Mayo Clinic’s Institutional Animal Care and Use Committee (IACUC Protocol# A00001864-16).

### Subject codes

Acellular, tissue engineering, extracellular matrix, mechanical behavior, mesenchymal stem cells, heart valve, pericardial, Transcatheter.

## Results

### Macroscopic tissue analysis

**Figure 1:**
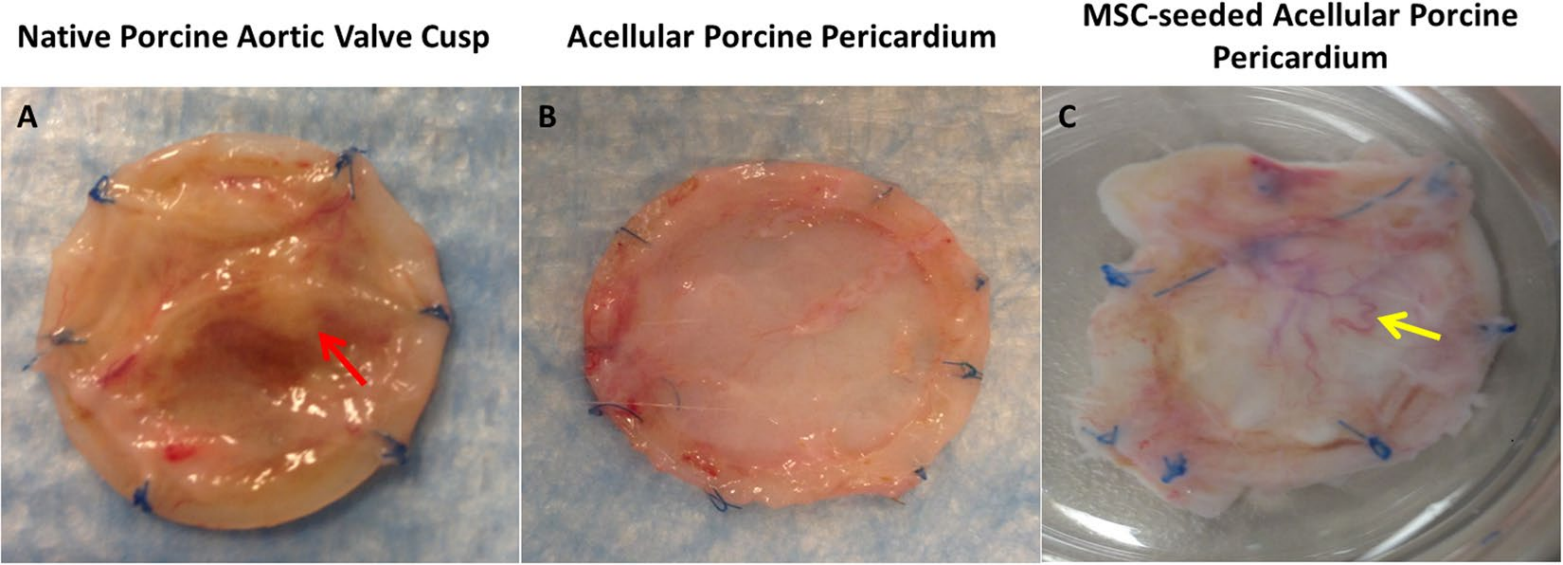
Macroscopic examination of tissue scaffolds. (**A**) Native porcine aortic valve cusps show severe inflammation (red arrow). (**B**) Acellular porcine pericardium showing no signs of inflammation. (**C**) MSC-seeded acellular porcine pericardium showed an area of neovascularization after 8 weeks (yellow arrow).

All the animals survived without evidence of sepsis or wound infection throughout the duration of the study. At all three time points, implanted native porcine aortic valve cusps expressed severe inflammation (Fig. 1). After 1 week, the acellular porcine pericardium was adhered to the adjoining muscle; however, after 8 weeks, tissue was easily separated by the host tissue. An area of neovascularization was observed in MSC-seeded acellular porcine pericardium after 8 weeks (Fig. 1).

### Transmission electron microscopy (TEM)

**Figure 2:**
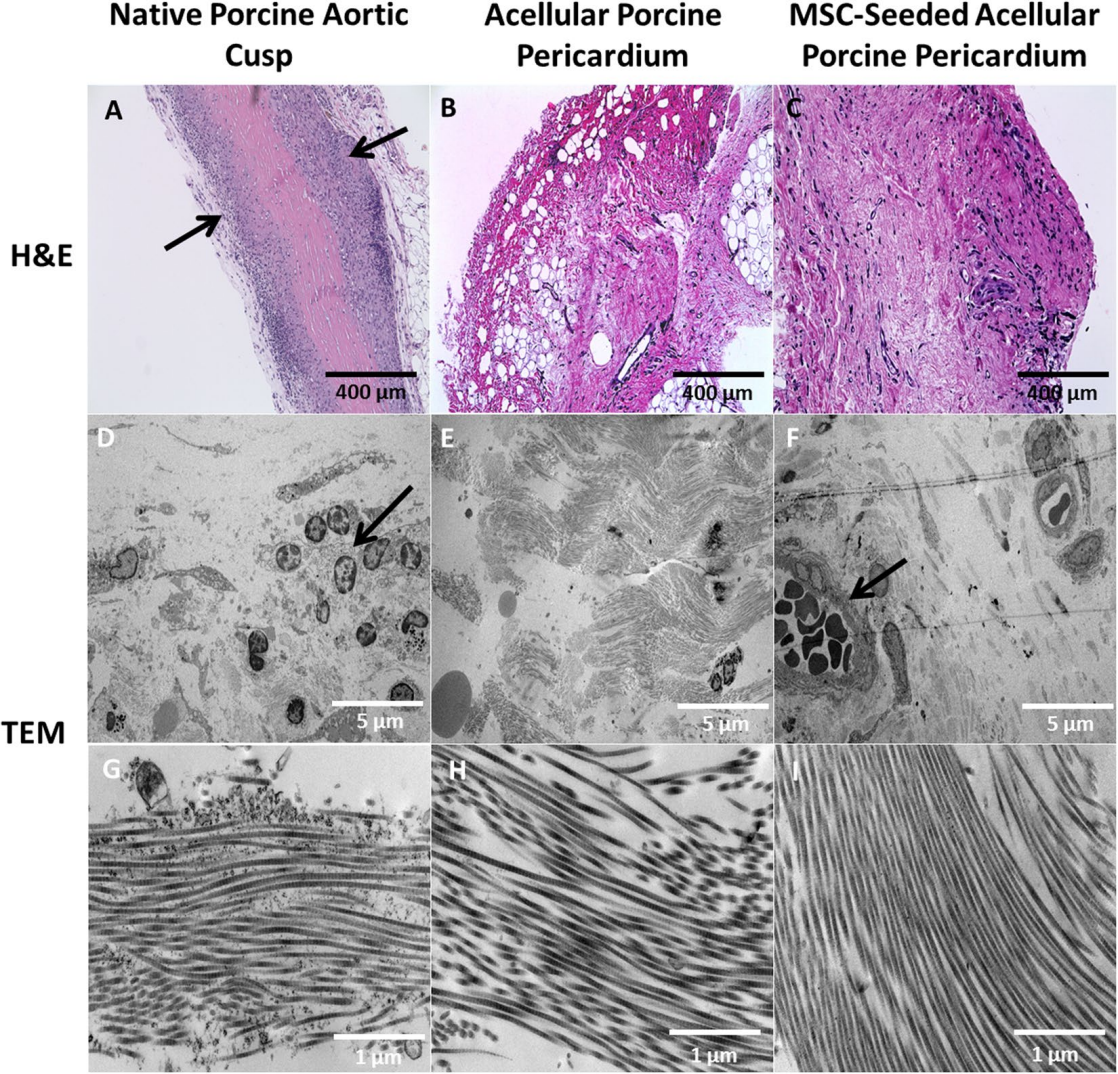
Inflammation, recellularization, and neovascularization of pericardium and cusp after 8 weeks. Hematoxylin and Eosin (400 µm) showed inflammatory cell invasion of the native aortic cusp (**A**) along the periphery (arrow). Progressive recellularization and neovascularization was observed in (**B**) acellular pericardium and (**C**) MSC-seeded pericardium. TEM (5 µm) analysis showed macrophage infiltration (arrow) observed in (**D**) native cusp. (**F**) Blood vessels (arrow) containing red blood cells were observed in MSC-seeded pericardium. TEM (1 µm) showed uniformity of collagen fiber architecture in all samples (**G–I**) with mild ECM distortion.

There was proinflammatory macrophage infiltration observed in the native cusps as observed on TEM imaging (Fig. 2). Additionally, neovascularization containing red blood cells were observed in MSC-seeded pericardium. Extracellular matrix evaluation with TEM revealed that the native porcine aortic valve cusps showed increased levels of inflammatory cell infiltration at 1 and 4 weeks with neovascularization that contained circulating macrophages and neutrophils (Fig. 2). Collagen architecture remained intact for both acellular porcine pericardium and MSC-seeded acellular porcine pericardium at all time points. MSC-seeded porcine pericardium showed faster onset of blood vessel formation after 4 weeks.

### Histology and immunohistochemistry

**Figure 3:**
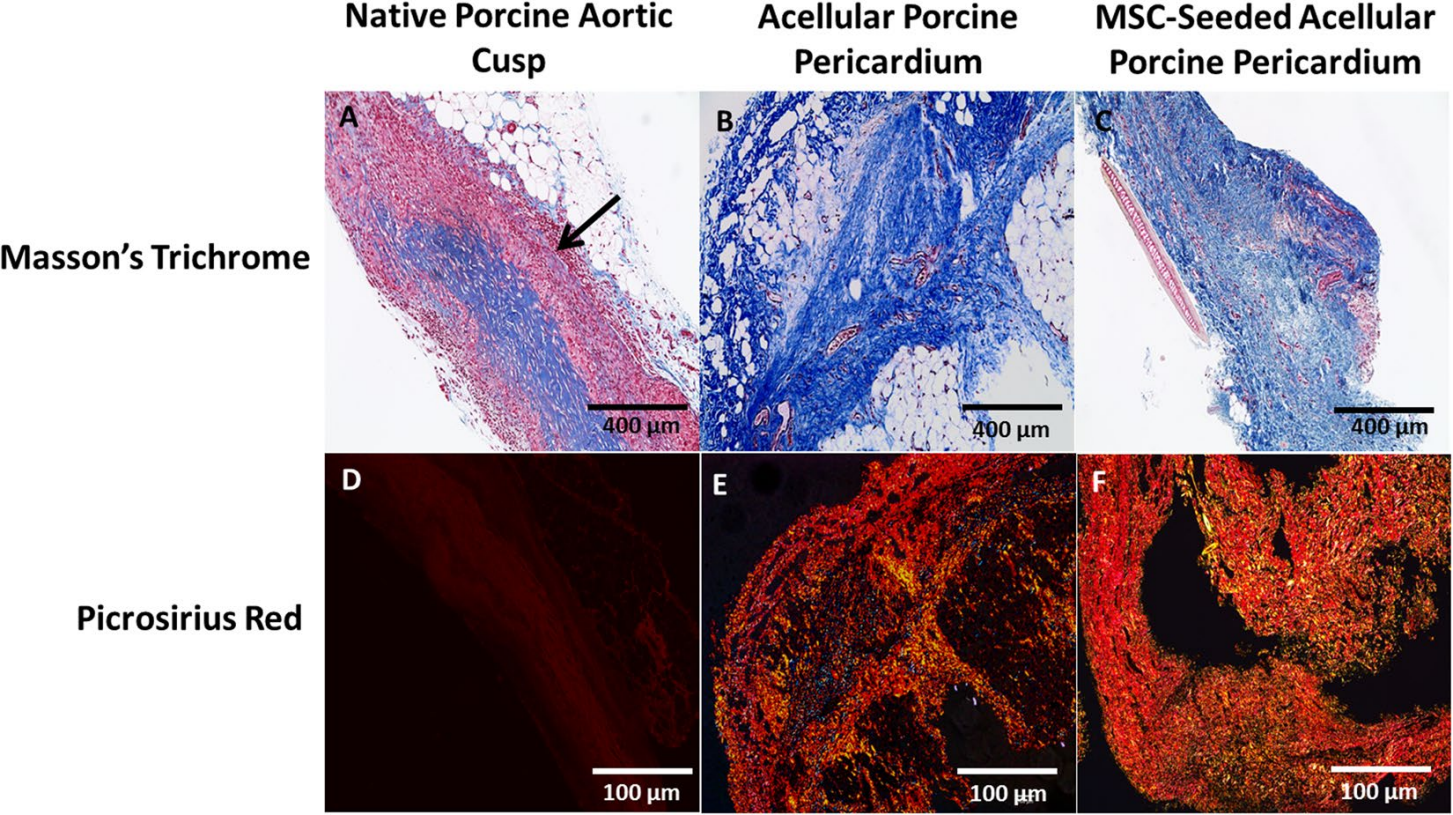
Masson’s Trichrome and picrosirius red staining confirmed collagen deposition in pericardium and cusp after 8 weeks. Collagen was evenly distributed in all pericardial samples; however, there was an observable increase of muscle fibers in the (**A**) native porcine aortic cusp (arrows). Collagen denoted with blue staining was appreciated in the (**A**) native cusp, (**B**) acellular pericardium, and (**C**) MSC-seeded pericardium.

Prior to implantation, the acellular porcine pericardium showed no cellular presence and this was confirmed by utilizing H&E and DAPI stains. At each time point, there was an observable amount of cell infiltration and acute and chronic inflammatory cells that surrounded the preexisting vasculature and periphery of the native porcine aortic valve cusps. While H&E staining showed invasion of these cells and distortion of the ECM in the native porcine aortic valve cusps (Fig. 2), there was progressive recellularization and neovascularization in the acellular and MSC-seeded porcine pericardium over time. Additionally, Masson’s Trichrome and Picrosirius Red staining showed that acellular and MSC-seeded porcine pericardial samples had higher collagen content compared to the native porcine aortic valve cusps (Fig. 3). Immunohistochemical (IHC) staining assessed the presence of M2 macrophages, angiogenesis, inflammation, and tissue recellularization.

### CD3/CD163

**Figure 4:**
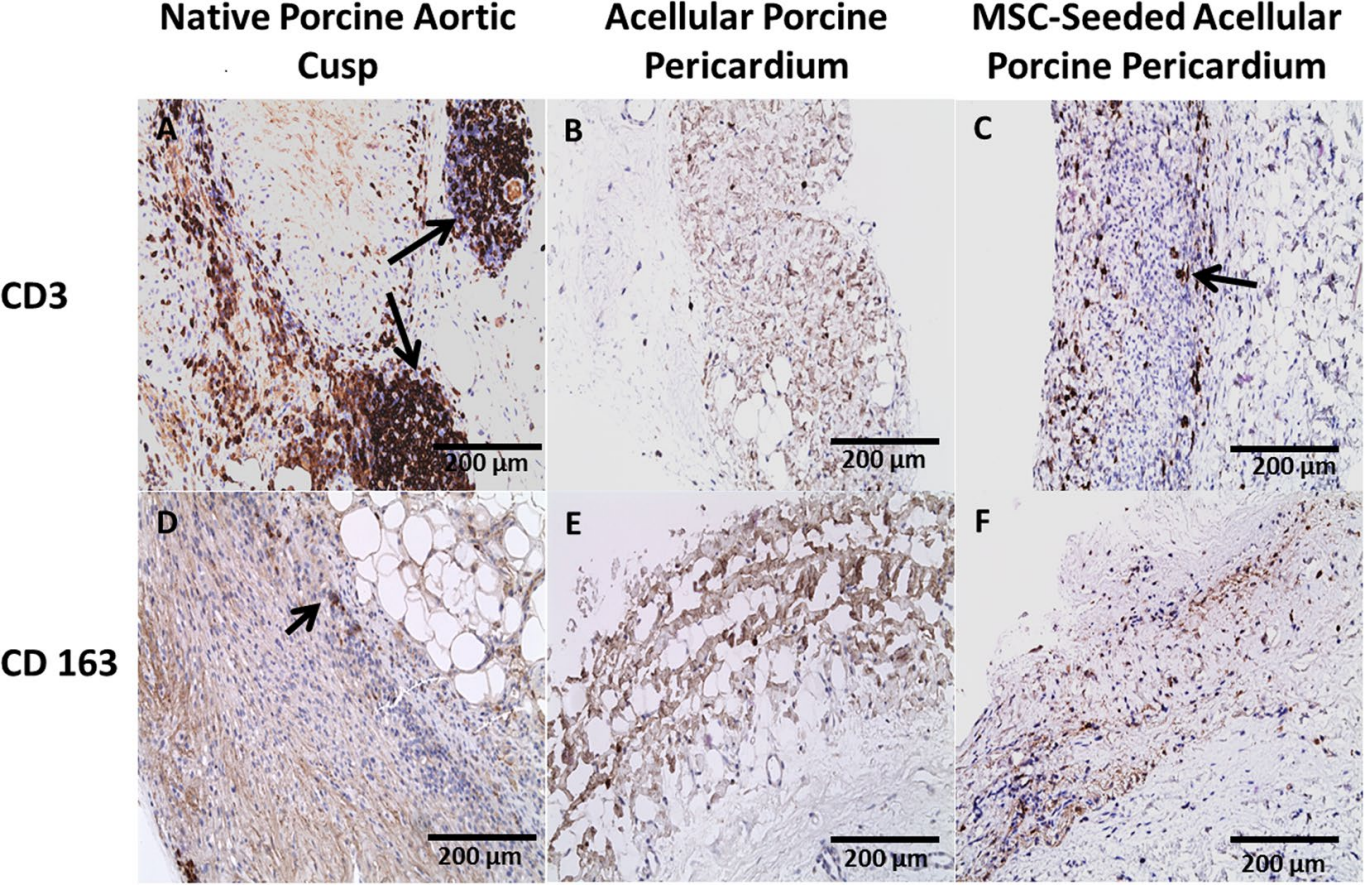
Immunohistochemistry for CD3 and CD163 after 8 weeks. Severe inflammation was observed in (**A**) native cusp (arrow), mild inflammation in (**C**) MSC-seeded pericardium (arrow), and no inflammation observed in (**B**) acellular pericardium. CD 163 staining specific marker of M2 macrophages showed increased expression in (**D**) native porcine aortic cusps (arrow) compared to (**E**) acellular porcine pericardium and (**F**) MSC-seeded pericardium.

A statistically significant difference was observed with CD3 showing an inflammatory reaction in native porcine aortic valve cusps compared to acellular and MSC-seeded porcine pericardium at all time points (P < 0.001) (Fig. 4). At 8 weeks, CD3 quantification for native porcine aortic valve cusps were found to be increased (13.43 ± 1.00) in contrast to acellular porcine pericardium which was weakly positive (0.18 ± 0.02). Despite this finding, the MSC-seeded porcine pericardium showed increased inflammation compared to acellular porcine pericardium, but significantly less than native porcine aortic valve cusps at the same time point (2.08 ± 0.01). A marker of M2 macrophages, CD163 was weakly expressed in all 3 groups at 1 week; however, acellular and MSC-seeded porcine pericardium had increased expression after 4 and 8 weeks (Fig. 4). All groups had a statistically significant increase in CD163 at 8 weeks compared to 1 week (P < 0.001) (Table 1).

**Table 1:** Statistical comparisons of semi quantative analysis for various IHC stains for native cusps, acellular, and recellularized pericardium between weeks 1, 4, and 8.

Group Comp Week 1	CD 3	CD 31	CD 34	CD 163	αSMA	Vimentin
Native Cusps vs Acellular Pericardium	<0.001*	<0.001*	<0.001*	0.035*	<0.001*	<0.001*
Native Cusps vs Recellularized Pericardium	<0.001*	<0.001*	<0.001*	0.017*	<0.001*	<0.001*
Acellular Pericardium vs Reseeded Pericardium	0.397	0.484	<0.001*	0.810	0.148	0.976
**Group Comp Week 4**						
Native Cusps vs Acellular Pericardium	<0.001*	<0.001*	<0.001*	0.934	<0.001*	<0.001*
Native Cusps vs Reseeded Pericardium	<0.001*	<0.001*	0.014*	0.096	<0.001*	<0.001*
Acellular Pericardium vs Reseeded Pericardium	<0.001*	<0.001*	0.006*	0.617	0.994	<0.001*
**Group Comp Week 8**						
Native Cusps vs Acellular Pericardium	<0.001*	<0.001*	0.033*	<0.001*	0.0018	<0.001*
Native Cusps vs Reseeded Pericardium	<0.001*	<0.001*	1.000	<0.001*	<0.001*	<0.001*
Acellular Pericardium vs Reseeded Pericardium	0.016*	<0.001*	0.033*	<0.001*	0.050*	<0.001*

Comparisons were performed with a one way ANOVA followed by a multiple comparison test. P value less than 0.05 shown with an asterisk.

### CD 31/CD34

**Figure 5:**
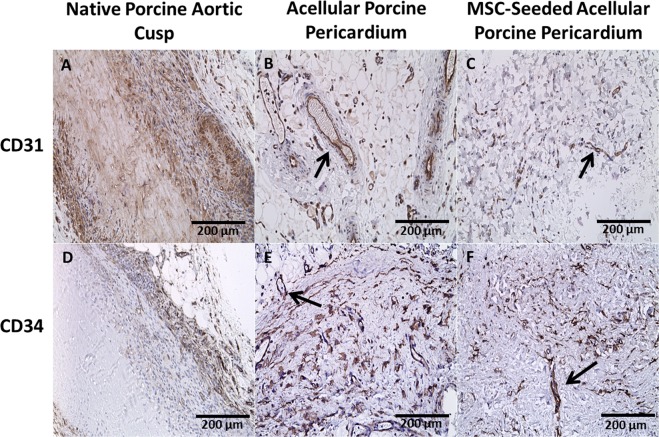
Immunohistochemistry for CD31 and CD34 after 8 weeks. CD 31 staining showed progressive angiogenesis in both acellular (**B,E**) and MSC-seeded (**C,F**) samples (arrows) over the time. CD 34 staining was progressively positive in all samples which showed progressive angiogenesis in all scaffolds.

CD 31, a marker for endothelial cells, revealed an increased expression for all samples after 8 weeks compared to 1 week (P < 0.001); however, there was a statistically significant difference observed in the native porcine aortic valve cusps compared to the acellular and MSC-seeded porcine pericardium (P < 0.03) (Fig. 5). The native porcine aortic valve cusps had the highest expression of CD31 with 6.92 ± 0.06 (1 week), 16.20 ± 0.2 (4 weeks), and 12.51 ± 1.01 (8 weeks). A marker for angiogenesis, CD 34 showed a statistically significant increase in expression at 8 weeks compared to 1 week for all tissue groups (P < 0.002), (Fig. 5).

### αSMA/Vimentin

**Figure 6:**
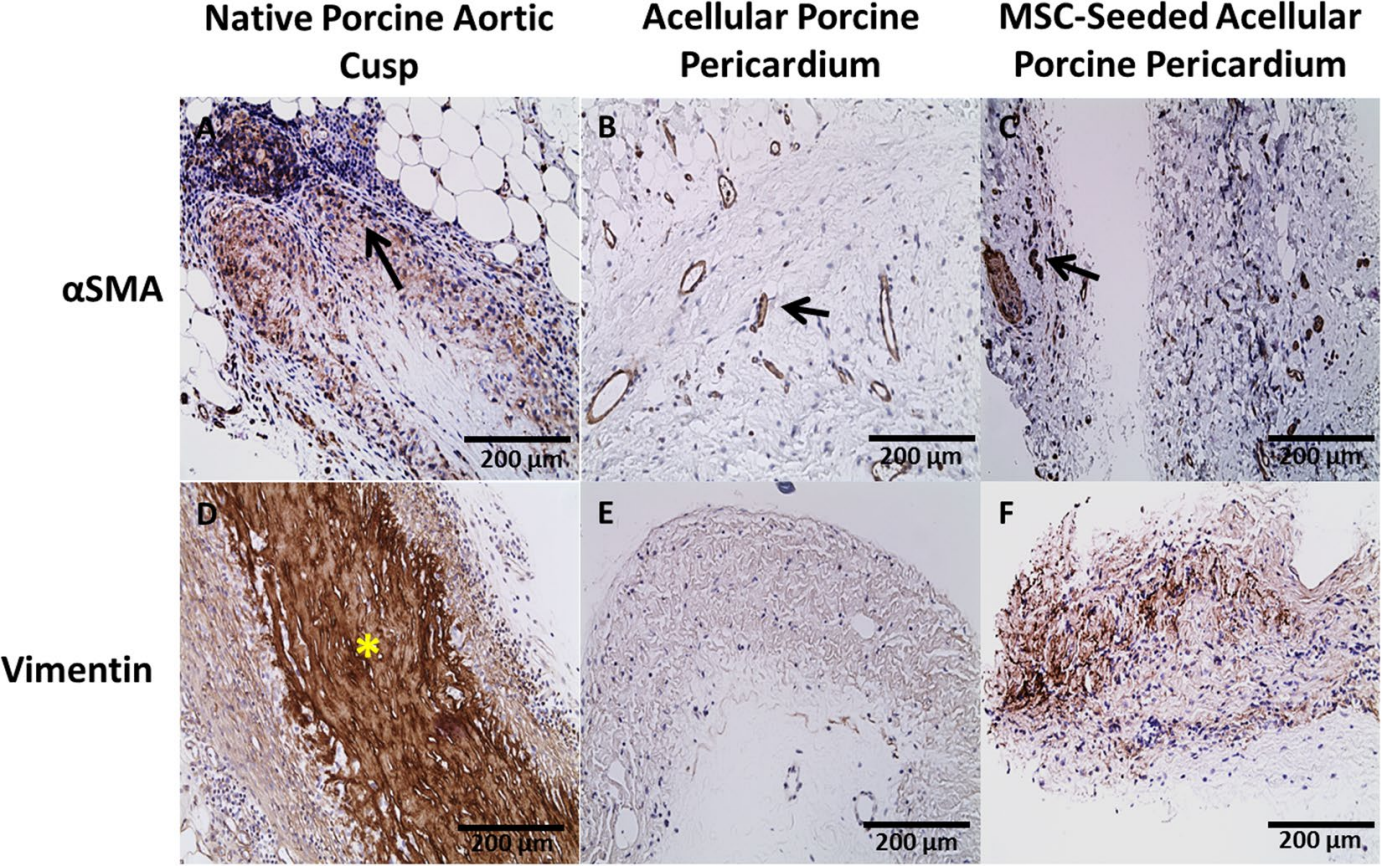
Immunohistochemistry for αSMA and Vimentin After 8 Weeks. Alpha smooth muscle was expressed in (**B**) acellular pericardium and (**C**) MSC-seeded pericardium. Vimentin positive cells were primarily present in (**D**) native cusp (yellow asterisk). The recruitment of vimentin positive cells started after 8 weeks in the (**E**) acellular and (**F**) MSC-seeded pericardium.

Vimentin was expressed in native porcine aortic valve cusps compared to acellular and MSC-seeded porcine pericardium (Fig. 6). The acellular and MSC-seeded porcine pericardium showed limited expression of vimentin at 1 and 4 weeks, but prominent expression at 8 weeks. Alpha smooth muscle actin staining showed increased expression of native porcine aortic valve cusps and acellular porcine pericardium throughout the entirety of the study (Fig. 6). There was a statistically significant increase in recellularization of the acellular porcine pericardium over the time (P < 0.001).

### Biomechanical properties: uniaxial tensile testing

**Figure 7:**
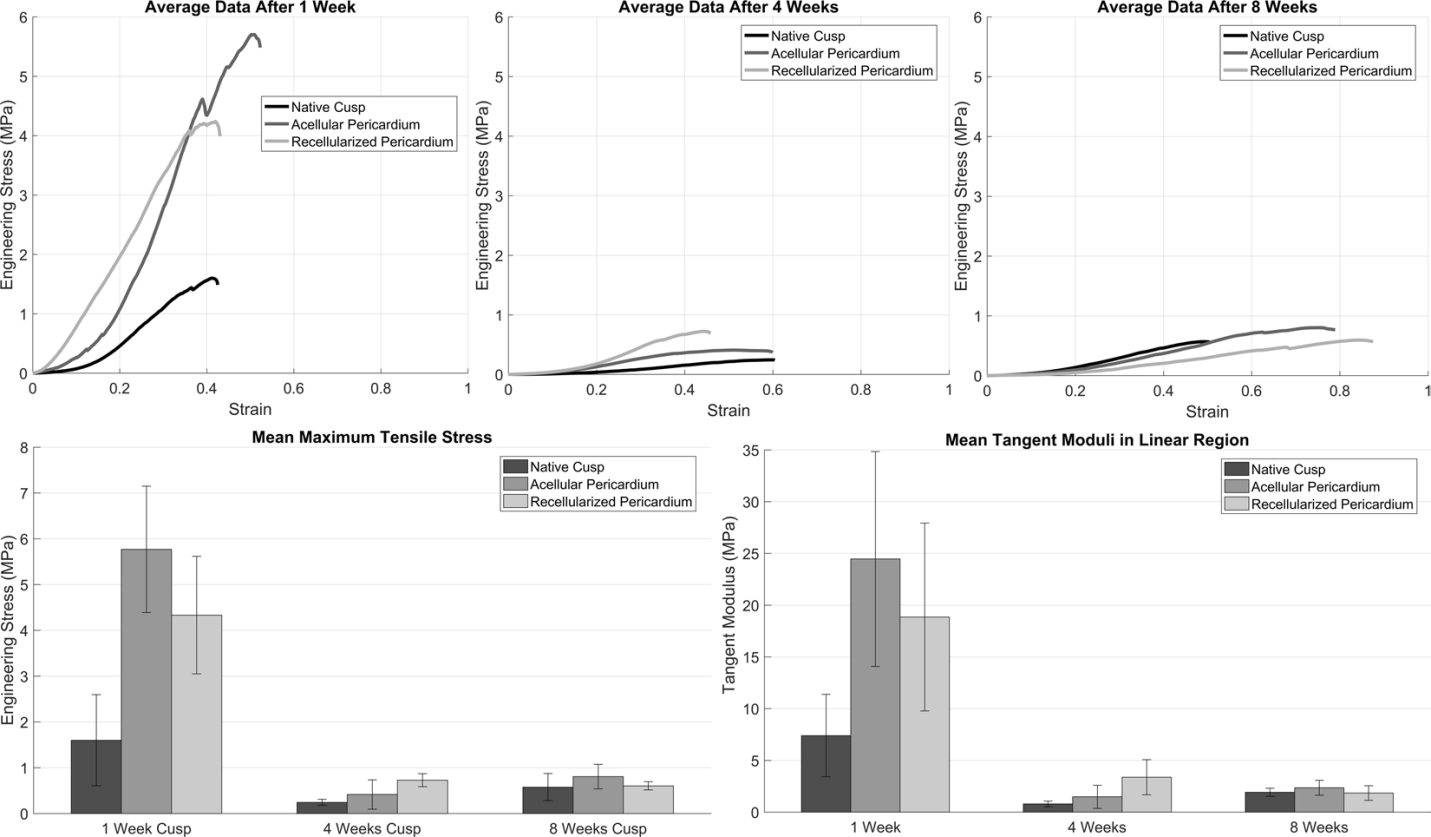
Biomechanical testing. **Top**. Engineering stress versus strain curves of the native cusps (C) at 1, 4 and 8 weeks subcutaneous implantation in rats. **Bottom**. Bar charts of the mean tensile stress and tangent modulus with error bars for acellular pericardium (P) at 1, 4 and 8 weeks subcutaneous implantation in rats. Significant differences between different tissues at the respect weeks is indicated with a star. There was a significant difference in both tensile stress and tangent modulus between 1 and 4 weeks but not 4 and 8 weeks.

Engineering stress versus strain plots are shown in Fig. 7 for each group at designated explant times. Overall, each curve had similar profile characteristics of soft tissues although respective stiffness and maximum stresses vary greatly (Fig. 7). Statistical comparisons between groups and explant times are shown in Tables 2 and 3. Maximal tensile stress at 1 week was 5.77 ± 1.38 and 4.33 ± 1.28 (MPa) for acellular porcine pericardium and MSC-seeded porcine pericardium; respectively. The tensile stress was significantly decreased at 4 weeks to 0.42 ± 0.32 and 0.73 ± 0.14 (MPa) for acellular (P = 2.077E-5) and MSC-seeded porcine pericardium; respectively (P = 0.0068) (Table 3). At 8 weeks a mild increase in tensile stress was seen with all samples (0.81 ± 0.27 and 0.6 ± 0.09 MPa for acellular and MSC-seeded porcine pericardium; respectively).The average maximum tensile stress at 1 week was 1.6 ± 1.0 (MPa) for native aortic valve cusps. Similar to pericardium, the tensile stress for cusps decreased at 4 and 8 weeks. However, these findings were not statistically significant, 0.25 ± 0.07 (MPa) at 4 weeks and 0.58 ± 0.3 at 8 weeks for native porcine aortic valve cusps. Thus, the addition of MSC seeded cells to an acellular pericardial scaffold does not offer a significant biomechanical advantage.

**Table 2:** Statistical comparisons of fracture stress and tangent modulus for native, acellular and recellularized tissue between weeks 1 and 4, weeks 1 and 8, and weeks 4 and 8.

	Native Cusps		Acellular Pericardium		Recellularized Pericardium	
p value	Fracture Stress	Tangent Modulus	Fracture Stress	Tangent Modulus	Fracture Stress	Tangent Modulus
Week 1–Week 4	0.076	0.031*	2.077E-5*	0.0012*	0.0068*	0.0506*
Week 1–Week 8	0.183	0.662	3.839E-5*	0.0015*	0.0095*	0.0537*
Week 4–Week 8	0.822	0.862	0.7892	0.9771	0.9848	0.9561

Comparisons were performed with a one way ANOVA followed by a multiple comparison test. p value less than 0.05 shown with an asterisk.

**Table 3:** Statistical comparisons of fracture stress and tangent modulus for native, acellular and recellularized cusps and pericardium between weeks 1 and 4, weeks 1 and 8, and weeks 4 and 8.

P value	Fracture Stress	Tangent Modulus	Fracture Stress	Tangent Modulus	Fracture Stress	Tangent Modulus
Native Cusp – Acellular Pericardium	0.003*	0.044*	0.605	0.737	0.511	0.655
Native Cusp – Recellularized Pericardium	0.045*	0.219	0.078	0.071	0.993	0.992
Acellular Pericardium – Recellularized Pericardium	0.325	0.657	0.235	0.156	0.654	0.639

Comparisons were performed with a one way ANOVA followed by a multiple comparison test. P value less than 0.05 shown with an asterisk.

## Discussion

The current study demonstrated that acellular and MSC seeded porcine pericardial scaffolds have good host recellularization. MSC seeded scaffolds facilitated a better host recellularization response, which may be useful for implementation in a TEHV. The current study found that acellular and MSC-seeded porcine pericardium showed (1) minimal inflammation, (2) better host cell recellularization, and (3) improved biomechanics, and therefore the current acellular process may hold promise for tissue engineered TAVR applications.

### Host cell response to unprocessed native porcine aortic valve cusps

Inflammation is a pathophysiological response that comes from the introduction of a foreign-body into a homeostatic environment. Chronic inflammation was observed utilizing the biomarker CD3. In native porcine aortic valve cusps, there was increased expression of the CD3 biomarker with infiltration of lymphocytes, macrophages, and blood vessel proliferation^26–28^. In studies done by Christ *et al*., a similar inflammatory foreign-body response was observed at the edge of native aortic valves in a rat subdermal implantation model^29^. Cellular remnants in the native porcine aortic valve cusps may have elicited a foreign-body reaction. Comparatively, acellular and MSC-seeded porcine pericardium elicited a minimal inflammatory response. While decellularization may alter the ECM, literature suggests that inflammation may have a direct role to decreased collagen crosslinking^30,31^. The current study utilized chemical decellularization to remove native cellularity, ultimately eliminating surface antigens that are confirmed with H&E and DAPI. However, Badylak *et al*. stated that decellularization does not completely eliminate foreign-body antigens that activate T-cells, accumulate polymorphonuclear cells, or lead to macrophage and/or monocyte infiltration^32–35^. Minimal expression of the CD3 biomarker was observed in the acellular and MSC-seeded porcine pericardium with the latter having the mildest expression. The MSCs that were utilized were collected from the same species, which was important because these cells have low immunogenicity and decreased expression of MHC I levels. This may explain the minimal inflammation observed in the MSC-seeded porcine pericardium^21,36^. Huang *et al*. referred to low immunogenicity of undifferentiated MSCs in the early phase of implantation; however, when differentiated these cells expressed increased MHC I levels. The immunomodulatory capability of MSCs was explained by allogenic T cell activation that induces little rejection^32,33,37^. Macrophage phenotype is divided into two categories; macrophage type I (M1) has a proinflammatory response and macrophage type 2 (M2) has an anti-inflammatory response with regenerative capacity^34,35,38,39^. The biomarker CD163 determines populations of the M2 phenotype, which were increasingly expressed in both acellular and MSC-seeded porcine pericardium. Expression of CD163 indicates regenerative capacity, and there was increased expression in MSC-seeded acellular porcine pericardium compared to acellular porcine pericardium alone after 8 weeks. This further emphasizes the role MSCs have in homing and differentiation of autologous cells through a paracrine secretion of growth and chemotactic factors that aid in regeneration^40–43^. Similar findings were reported by Sayk *et al*. and Kallyne *et al*. where acellular porcine dermal tissue expressed both an M1 response at 14 days and an M2 response after 21 days. In the current study, a delayed response in the M2 population was increasingly observed between 4 and 8 weeks^44^.

### Recellularization of acellular and MSC-seeded acellular porcine pericardium

In all groups, CD 31 and CD 34 biomarkers were expressed; however, increased expression was appreciated more in the acellular and MSC-seeded porcine pericardial groups. These findings strongly suggest that there was increased endothelialization and angiogenesis. The native porcine aortic cusps are naturally encompassed by an endothelial layer that is independent from *in vivo* recellularization. However, in the acellular and MSC-seeded groups, there was increased recruitment of angiogenic factors leading to increased endothelialization. Under transmission electron microscopy, the current study confirmed an increase presence of neovascularization that contained circulating red blood cells. In previous studies, decellularized tissues have been known to contribute to cell migration, proliferation, and differentiation^45^. Ingber *et al*. mentioned that neovascularization contains erythrocytes within the lumen indicating a functional connection with the host vascular system^46^. Many studies have compared MSC-seeded tissue in an *in vivo* and *in vitro* environment to determine the effects of seeding on acellular tissue^47,48^. In the current study, the presence of alpha-SMA and vimentin increased after 8 weeks in both the acellular and MSC-seeded porcine pericardium. This finding suggests that there may be a phenotypic modulation of resting fibroblast-like cells to a more active form of myofibroblast-like cells that mediates connective tissue remodeling and formation of new collagen. In addition to regenerative capacity, the collagen content and biomechanical properties were evaluated. It may be speculated that the *in vivo* environment activated interstitial-like cells to create new collagen fibrils, which then impacted the mechanical properties. When comparing all three groups, the MSC-seeded scaffolds had better regenerative capacity and biomechanical properties. The results showed that the maximum tensile stress decreased significantly from 1 to 4 weeks in all groups, which may be due to resorption of foreign tissue before subsequent remodeling and protein fiber synthesis^49^. This theory is supported by the PicroSirius Red staining, which showed a decrease in collagen distribution for all groups.

### Biomechanical properties

The tensile stress showed no significant changes between 4 and 8 weeks post implantation. The underlying mechanism for deterioration of biomechanical properties could be addressed by Tohyama *et al*. who proposed that extrinsic cellular infiltration and revascularization from the surrounding tissues accelerate the deterioration of mechanical properties of a patellar tendon matrix^50^. Reduced mean maximum tensile stress and mean tangent modulus at week 1 for MSC seeded tissue compared to acellular porcine pericardium and native porcine aortic valve cusps. The MSCs may have hastened tissue resorption leading to a decrease in mechanical strength^51^. Additionally, MSCs could encourage deposition of material that does not give increased mechanical strength, but increased tissue thickness reducing the calculated stress. Despite superior results in the MSC-seeded group for vimentin and αSMA indicating that SMCs and VICs are present in the scaffold, it was observed that new fibers were not being synthesized to replace tissue that had been resorbed. The collagen content was significantly decreased in acellular porcine pericardium, MSC-seeded porcine pericardium, and native pericardium between 1 and 4 weeks; however, there was no significant changes after 4 weeks. The collagen content for native porcine aortic valve cusps decreased compared to acellular and MSC-seeded porcine pericardium. It may be speculated that the inflammation observed in the native porcine aortic cusps may have affected the collagen content. *Jetty et al*. demonstrated that after 4 weeks, the inflammatory response was severe that mild to moderate degradation of collagen fibers were observed in aortic graft samples. While Mimura *et al*. showed that acellular dermal matrix and native samples had similar collagen distribution after 3 days, but there was a significant decrease in collagen a couple weeks later^52^. Overall, there was no mechanical advantage for utilizing an MSC-seeded over an acellular scaffold. After *in vivo* characterization, both acellular and MSC seeded pericardial scaffolds had superior mechanical properties compared to native valve cusps.

### Limitations to model

The model chosen provides a simple, cost-effective means of evaluating the recellularization potential for the samples considered in this study. However, there are limitations to this model; the first of which is that this model does not expose the tissue to cells commonly found in the vascular system such as valvular interstitial cells and valvular endothelial cells thus the effect of the seeding MSCs here on the infiltration of these cell types can only be speculated. Additionally, the mechanical environment differs greatly to the vascular system in which tissue will be under pulsatile loading which will expose the tissue to different shear and normal stresses than in this model. This will be sensed by cells via mechanotransduction which may alter their behavior leading to different protein fiber synthesis and different mechanical response.

## Conclusion

The regenerative capacity of acellular and MSC-seeded porcine pericardium showed angiogenesis and regeneration of smooth muscle cell and macrophage recruitment. Although the native porcine aortic valve cusps showed recellularization, there was more inflammation compared to acellular and MSC-seeded porcine pericardium. Reseeding with MSCs had better regenerative outcomes compared to acellular scaffolds without cell seeding. The current study highlighted some challenges for tissue engineered scaffolds, specifically regarding the reduction of collagen content and deterioration during the early phase of implantation. The subcutaneous model was found to be a good model to evaluate the regenerative capacity for tissue engineered scaffolds, and provided baseline biomechanical behavior. Further studies will involve chronic animal studies that are exposed to physiological blood flow in a homeostatic environment.

## Data Availability

The datasets generated during and/or analyzed during the current study are available from the corresponding author on reasonable request.
